# Circular RNA F-circSR derived from *SLC34A2-ROS1* fusion gene promotes cell migration in non-small cell lung cancer

**DOI:** 10.1186/s12943-019-1028-9

**Published:** 2019-05-22

**Authors:** Ke Wu, Xun Liao, Youling Gong, Juan He, Jian-Kang Zhou, Shuangyan Tan, Wenchen Pu, Canhua Huang, Yu-Quan Wei, Yong Peng

**Affiliations:** 1 State Key Laboratory of Biotherapy and Cancer Center, National Clinical Research Center for Geriatrics, West China Hospital, Sichuan University, Chengdu, 610041 China; 2 Department of Thoracic Oncology and Cancer Center, West China Hospital, Sichuan University, Chengdu, 610041 China

**Keywords:** *SLC34A2-ROS1*, Circular RNA, Cell migration, NSCLC

## Abstract

Cancer-associated chromosomal translocations are reported to generate oncogenic circular RNA (circRNA), contributing to tumorigenesis. The fusion gene *SLC34A2-ROS1* (solute carrier family 34 member 2 and ROS proto-oncogene 1) plays an important role in non-small cell lung cancer (NSCLC) progression. However, whether *SLC34A2-ROS1* gene can produce circRNA remains unknown. Here, we identified two novel circRNAs (F-circSR1 and F-circSR2) generated from *SLC34A2-ROS1* fusion gene, while F-circSR1 has higher expression than F-circSR2. Functional studies through gain- and loss-of-function strategies showed that both F-circSRs promote cell migration in lung cancer cells, whereas they have little effect on cell proliferation. Using the minigene GFP reporter assay, we verified that the flanking complementary sequences with canonical splicing sites are essential for F-circSR biogenesis. Therefore, our findings demonstrate the oncogenic role of F-circSR in NSCLC and highlight its therapeutic potential.

**Electronic supplementary material:**

The online version of this article (10.1186/s12943-019-1028-9) contains supplementary material, which is available to authorized users.

## Main text

Non-small cell lung cancer (NSCLC) is the most common type of lung cancer worldwide, accounting for approximately 85% of lung cancers [1]. Despite achievements in clinical diagnosis and treatment, NSCLC patients have poor survival. Therefore, a better understanding of molecular mechanisms underlying NSCLC could promote discovery of novel therapeutic targets and improve survival.

A subtype of NSCLC harbors *ROS1* fusion genes from aberrant chromosomal translocations of two separated genes. Among them, *SLC34A2-ROS1* fusion gene encodes oncogenic fusion protein to activate downstream signaling cascades such as JAK/STAT, PI3K/Akt and RAS/RAF pathways and promote cell proliferation and survival [2]. However, the underlying mechanism of *SLC34A2-ROS1* gene during tumorigenesis remains unclear.

Emerging evidences demonstrated that, except for encoding fusion protein, fusion gene could generate circular RNA (circRNA), a covalently-bonded RNA molecule from the back-splicing of linear RNA, to participate in tumor initiation and progression. For example, *PML-RARα* fusion gene produces f-circPR to promote cell growth in acute promyelocytic leukemia, while *MLL-AF9* fusion gene generates f-circM9 that contributes to leukemia progression in vitro and in vivo [3]. Recently, we found that *EML4-ALK* fusion gene can produce two circRNAs [4, 5], one of which is a novel liquid biopsy biomarker for lung cancer [5]. However, whether *SLC34A2-ROS1* gene generates circRNA is unknown. In this study, we identify the novel circRNA F-circSRs generated from *SLC34A2-ROS1* gene. Moreover, F-circSRs, independent from *SLC34A2-ROS1* fusion protein, have little effect on cell proliferation, but promote cell migration in lung cancer cells, highlighting the oncogenic role of F-circSRs in NSCLC.

## Results and discussion

### Identification of F-circSR in NSCLC cells

**Fig. 1:**
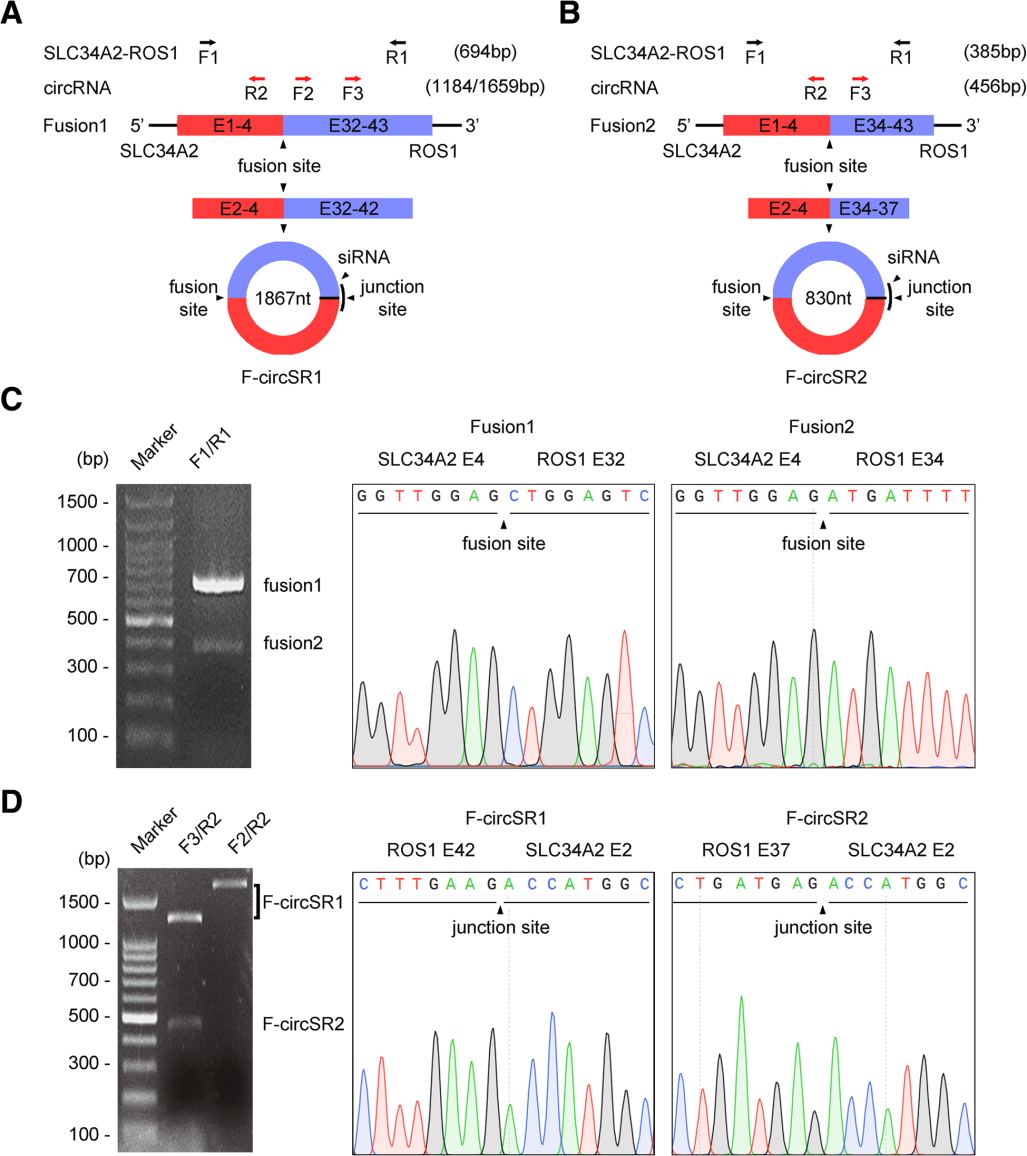
Identification of F-circSR in HCC78 cells. **a~b** Schematic representation of F-circSR generated from *SLC34A2-ROS1* fusion gene. E means exon. **c** Agarose gel electrophoresis and Sanger sequencing of RT-PCR products from HCC78 cells, the arrows indicate *SLC34A2-ROS1* fusion sites. **d** Identification of F-circSR in HCC78 cells by RT-PCR and Sanger sequencing. The arrows indicate F-circSR junction sites

It is reported that the NSCLC cell line HCC78 harbors two forms of *SLC34A2-ROS1* fusion genes expressing long and short transcripts (*SLC34A2* exon 4 fused to *ROS1* exons 32 and 34, respectively) (Fig. 1a, b). To verify this, we performed RT-PCR using the convergent primers (F1/R1, Additional file 1). The Sanger sequencing results confirmed the existence of two *SLC34A2-ROS1* mRNA variants in HCC78 cells, same as the reference sequences EU236946.1 and EU236947.1 in GenBank (Fig. 1c). Next, we investigated whether *SLC34A2-ROS1* fusion genes generate circRNA. Total RNAs extracted from HCC78 cells were treated with RNase R to remove linear RNAs, and then subjected to RT-PCR using the divergent primers (F2/R2 and F3/R2, respectively). As shown in Fig. 1d, two circRNAs, designated as F-circSR1 (from longer variant) and F-circSR2 (from shorter variant), were identified by agarose electrophoresis and Sanger sequencing because of the existence of the back-splicing junction between the 5′ head of *SLC34A2* exon 2 and 3′ tail of *ROS1* exon 37 or 42. Moreover, qPCR results showed that F-circSR1 has much higher expression level than F-circSR2 in HCC78 cells (Additional file 2: Figure S1). Therefore, these data indicate *SLC34A2-ROS1* fusion gene generates two circRNAs in HCC78 cells, with higher F-circSR1 expression.

### F-circSRs promote cell migration in NSCLC cells

**Fig. 2:**
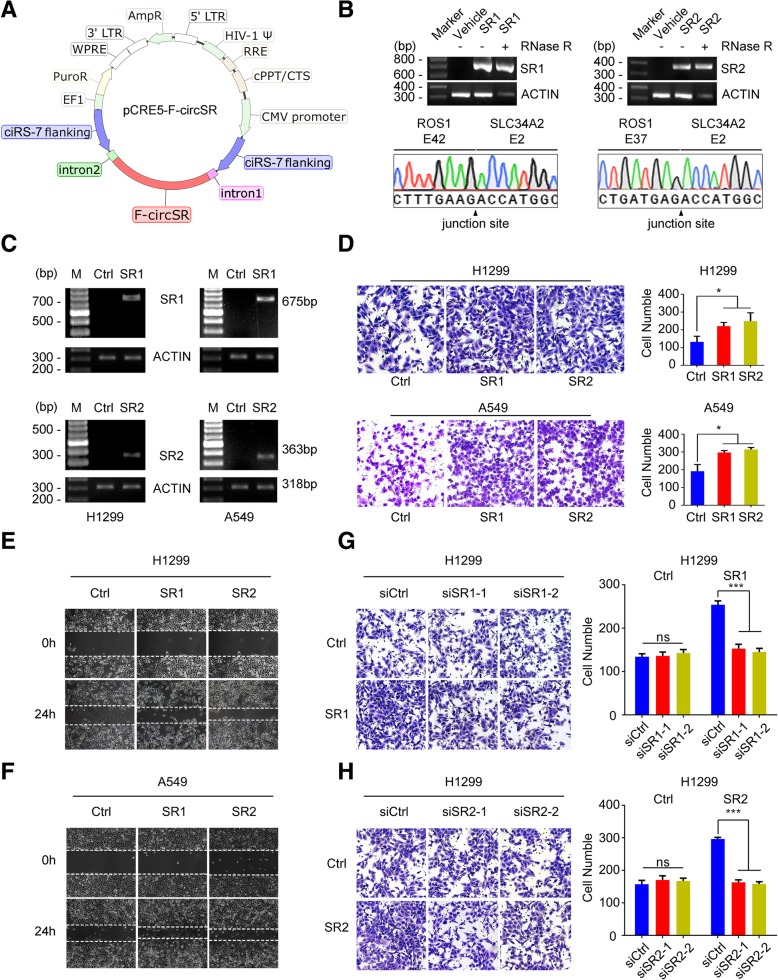
F-circSR promotes cell migration in NSCLC cells. **a** Schematic representation of pCRE5-F-circSR-expressing plasmid. **b** Agarose gel electrophoresis and Sanger sequencing of RT-PCR products from HEK293T cells transfected with F-circSR-expressing plasmid or empty vector. **c** Agarose gel electrophoresis of RT-PCR products from A549 and H1299 cells stably expressing F-circSR. **d** Representative images of Transwell migration assays and quantification in A549 and H1299 cells with or without F-circSR overexpression. **e~f** Representative images of wound healing assays in H1299 and A549 cells with or without F-circSR overexpression. **g~h** F-circSR knockdown attenuates the migratory ability in F-circSR1 (g) or F-circSR2 (h) overexpressing H1299 cells. SR1: F-circSR1; SR2: F-circSR2; E: exon

To characterize biological function of F-circSR in NSCLC cells, we constructed F-circSR-overexpressing plasmids with reverse repeat of ciRS-7 intron sequences plus the upstream and downstream flanking intron sequences of F-circSR, which favor circRNA formation (Fig. 2a). Firstly, the F-circSR-overexpressing plasmids were transiently transfected into HEK293T cells. The successful expression of both F-circSR1 and F-circSR2 and their accurate circularization were confirmed by agarose electrophoresis and Sanger sequencing (Fig. 2b). Then we chose H1299 and A549 cells to generate stable cells correctly expressing F-circSRs because both cells don’t express *SLC34A2-ROS1* fusion protein (Fig. 2c, Additional file 2: Figure S2a). Transwell migration assays show that both F-circSRs significantly promoted cell migration in both cells (Fig. 2d), which were further confirmed by wound healing assays (Fig. 2e, f). However, MTT assays and colony formation experiments indicated that both F-circSRs have little effect on cell proliferation (Additional file 2: Figure S2b, S2c). To avoid the bias caused by circRNA expression plasmid, we employed our previously reported expression system [4]. And the results confirmed again that F-circSRs promote cell migration in NSCLC cells (Additional file 2: Figure S3).

Besides gain-of-function experiments, the loss-of-function strategy was also used to examine cellular function of F-circSR. The small interfering RNAs (siRNAs) targeting the back-splicing junction of circRNAs were designed to efficiently knock down F-circSR expression (Additional file 2: Figure S2d, S2e). Transwell migration assays showed that silencing of F-circSR significantly decreased the cell migratory ability in F-circSR-overexpressing H1299 (Fig. 2g, h) and A549 cells (Additional file 2: Figure S2f-S2 h), convincing the role of F-circSR in promoting cell migration in NSCLC cells.

It is well documented that *SLC34A2-ROS1* fusion gene encodes the fusion protein to participate in tumorigenesis by activating ROS1 signaling [2]. Our findings suggest that except for encoding fusion protein, *SLC34A2-ROS1* fusion gene may exert its oncogenic role through generating circRNAs. Moreover, we performed bioinformatics analysis to find that F-circSRs harbor the binding sites of miR-150-5p, miR-194-3p and miR-515-5p that have been reported to regulate cell migration. So F-circSR may act as miRNA sponge to exert its function (Additional file 2: Figure S4). Giving that circRNA could be a potential biomarker due to its higher stability in body fluids, the diagnostic potential of F-circSR for lung cancer needs further investigation.

### The complementary sequences in flanking introns are important for F-circSR biogenesis

**Fig. 3:**
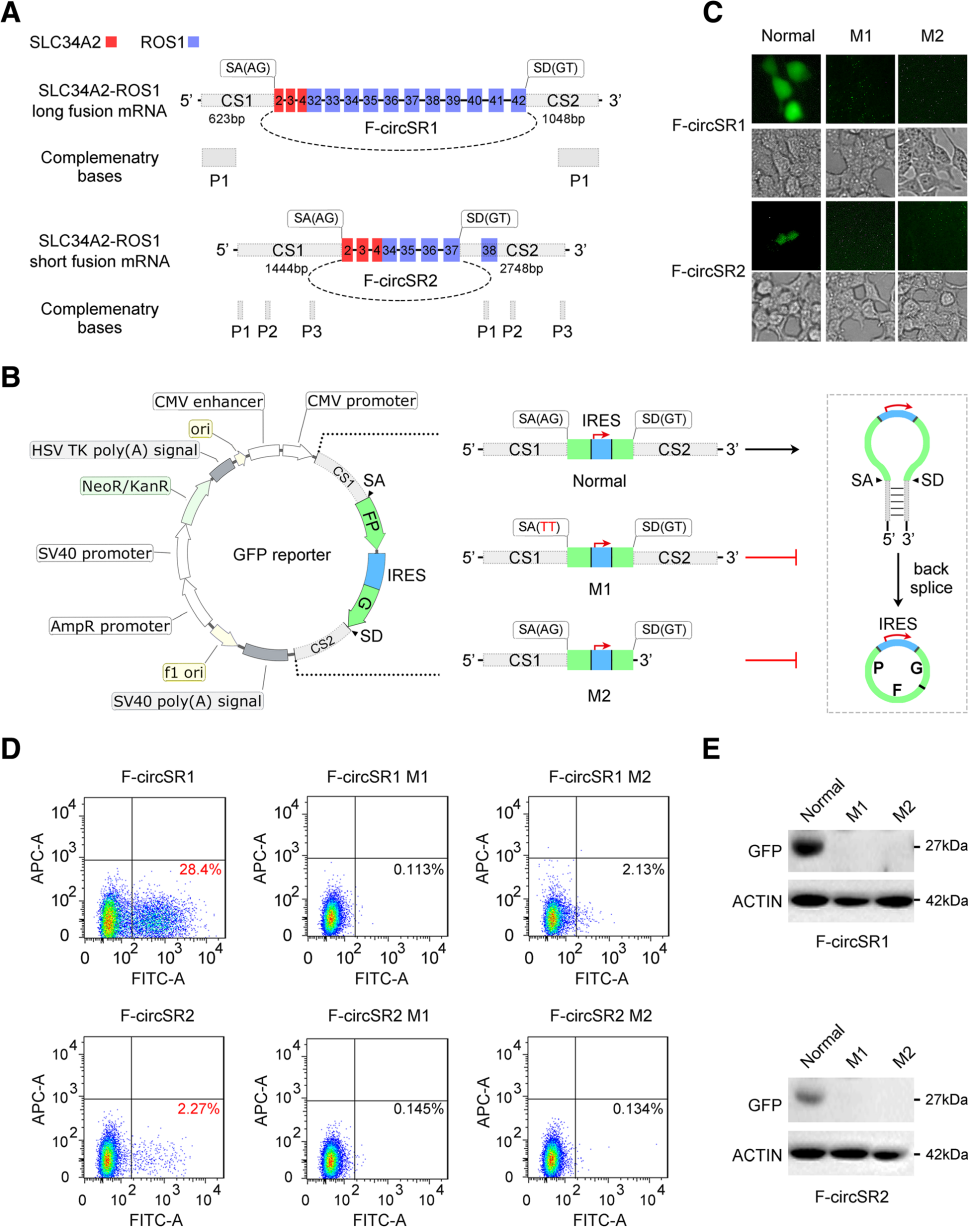
The flanking complementary sequences are important for F-circSR biogenesis. **a** Bioinformatics analysis of the cis-elements in the flanking introns of F-circSR. CS: upstream and downstream sequences of F-circSR; SA, splicing acceptor; SD, splicing donor. The circle denotes the junction site. The grey boxes called P indicate complementary sequences. **b** Schematic representation of GFP-based reporters to examine essential elements for circRNA formation. **c~e** GFP detection through fluorescence microscope **(c)**, flow cytometry **(d)** and Western blot **(e)**

Similar to most circRNAs, both F-circSRs consist of multiple exons. CircRNA biogenesis depends on the cis-regulatory elements that reside in the flanking introns of circularized exons, usually containing reverse complementary sequences [6, 7]. Bioinformatics analysis revealed that the flanking introns of both F-circSRs have reverse complementary sequences (designated as CS1 and CS2), while the CS1-CS2 complementarity in F-circSR1 is stronger than that in F-circSR2 due to the longer matching sequence (Fig. 3a, Additional file 3). Next we subcloned these sequences into the minigene GFP reporter system containing a single exon encoding split GFP in reverse order (Fig. 3b, Additional file 4), which does not express normal GFP protein unless inserted sequences back splice to produce a circRNA [8]. Additionally, we also constructed the plasmids with the mutation of splicing sites (from “AG” to “TT”, M1 plasmid) or deletion of the downstream complementary sequence (M2 plasmid) (Fig. 3b). As shown in Fig. 3c, inserted normal CS1 and CS2 sequences from either F-circSR can drive circRNA formation to express GFP protein, with the sequences from F-circSR1 showing stronger activity than those from F-circSR2, consistent with the higher CS1-CS2 complementarity in F-circSR1 from bioinformatics data. So stronger pairing of longer sequences considerably enhances circRNA production. However, destroying the splicing sites or complementary sequences of F-circSRs blocks circRNA formation, thus lacking GFP expression (Fig. 3c). These conclusions were further confirmed by Western blotting and flow cytometry analyses (Fig. 3d, e). Therefore, the flanking complementary sequences with canonical splicing sites are important for F-circSR formation.

## Conclusions

In this study, we identified two novel circRNAs (designated as F-circSR1 and F-circSR2) generated from *SLC34A2-ROS1* fusion gene in NSCLC cells, with higher expression of F-circSR1 than F-circSR2, whose formation depends on their flanking complementary sequences with canonical splicing sites. Moreover, both F-circSRs can significantly promote cell migration. Therefore, our study not only expands the current knowledge of chromosomal translocations in cancer biology, but also provides potential diagnostic and therapeutic biomarker.

## Additional files

Additional file 1: Information about primers, siRNAs and full sequence of F-circSR. (DOCX 40 kb)

Additional file 2: **Figure S1.** Absolute quantification of F-circSRs in HCC78 cells using qPCR. **Figure S2.** Characterization of cellular function of F-circSR in lung cancer cells. **Figure S3.** Validation of cellular function of F-circSR using pLaccase2 circRNA expression system. **Figure S4.** Predicted miRNA biding sites in F-circSRs. (DOCX 2870 kb)

Additional file 3: Bioinformatics analysis of the cis-elements in the flanking introns of F-circSR. (DOCX 1084 kb)

Additional file 4: Supplementary Materials and Methods. (DOCX 2750 kb)

## Abbreviations

circRNA: circular RNA
CS: complementary sequence
GFP: green fluorescent protein
NSCLC: non-small cell lung cancer
qPCR: real-time quantitative polymerase chain reaction
ROS1: ROS proto-oncogene 1
RT-PCR: reverse transcription polymerase chain reaction
SLC34A2: solute carrier family 34 member 2
