# Supplementary material for: Circular RNA F-circSR derived from SLC34A2-ROS1 fusion gene promotes cell migration in non-small cell lung cancer
Source: Mol Cancer. 2019 May 22;18:98. doi: 10.1186/s12943-019-1028-9 (PMC6530145; doi:10.1186/s12943-019-1028-9)
Supplement: Supplementary file 1 — Information about primers, siRNAs and full sequence of F-circSR. (DOCX 40 kb) [file 12943_2019_1028_MOESM1_ESM.docx]

| **Information about primers, siRNAs and full sequence of F-circSR** |
| --- |
| Primers for identification of *SLC34A2-ROS1* fusion gene  F1 GTGTGCTCCCTGGATATTCTTAG  R1 CCACTGCTGTTCCTTCATACA |
| Primers for identification of F-circSR1 and F-circSR2  F2 TCCTGAAGAGTGGGTAGGTT  R2 CAGTGGGAGAAAGCTGAAGATAA  F3 TTCCAACCCAAGAGGAGATTG |
| Primers for qPCR of F-circSR  F-circSR1  P1 TAGCATTTATAAGTCCAGAGATGAAGC  P2 AGGGAGCCATGGTCTTCAA  F-circSR2  P3 CTGAAGGAGGCACATCTGA  P4 CCCTTCGAGGTACTTATCGG |
| Primers for F-circSR1-overexpressing plasmid in pCRE5 vector  F-circSR1 1^st^ round:  P5 CCTTTCCATGACTGCTGCTTTAAGCTGTTTTCTCATCCACAGACCATGGCTCCCTGGCCTGA  P6 ACAGTGCAGCGAAAACTAGAAAATTCTGAAGAATCAAACTTACCTTCAAAGCTTTCATTTAT  F-circSR1 2^nd^ round:  P7 CAGATATCCATCACACTGGCGGCCGCGTGTGCCTCCTTTCCATGACTGCTGCTTTA  P8 TCCAGAGTAACCACTCGAGTCAGTTCACAGTGCAGCGAAAACTAGAAAA |
| Primers for F-circSR2-overexpressing plasmid in pCRE5 vector  F-circSR2 1^st^ round:  P5 CCTTTCCATGACTGCTGCTTTAAGCTGTTTTCTCATCCACAGACCATGGCTCCCTGGCCTGA  P9 ACTTCTTACCCATACCAGGAGAAAATTATTTCAGAGCTACCTCATCAGATGTGCCTCCTTCA  F-circSR2 2^nd^ round:  P7 CAGATATCCATCACACTGGCGGCCGCGTGTGCCTCCTTTCCATGACTGCTGCTTTA  P10 TCCAGAGTAACCACTCGAGCGGACTTAAAACTTCTTACCCATACCAGGAGAAAA |
| Primers for F-circSR1-overexpressing plasmid in pLaccase2 vector  P11 ATTTTTTTTATTTTATGCAGACCATGGCTCCCTGGCCT  P12 TTGGAATTTTGAATACTTACCTTCAAAGCTTTCATTTATGACTCCA |
| Primers for F-circSR2-overexpressing plasmid in pLaccase2 vector  P11 ATTTTTTTTATTTTATGCAGACCATGGCTCCCTGGCCT  P13 TTGGAATTTTGAATACTTACCTCATCAGATGTGCCTCCTTCA |
| Primers for semi-quantitative RT-PCR  F-circSR1  P14 GTGAAGATTGGAGACTTTGGAC  P15 TCCTGAAGAGTGGGTAGGTT  F-circSR2  P16 TGTATGAAGGAACAGCAGTGG  P15 TCCTGAAGAGTGGGTAGGTT  Actin  P17 ATCATGTTTGAGACCTTCAACA  P18 CATCTCTTGCTCGAAGTCCA |
| Primers for construction of GFP reporter plasmids  F-circSR1 CS1  P19 GAACCGTCAGATCCGCTAGCTTTTTTTTTTTTTTTGAGAAGGAGTTT  P20 TTGAAGAAGATGGTGCGCTCCTGTGGATGAGAAAACAGCTTAAAGC  Fragment of pCIRC-FP-IRES-G  P21 GAGCGCACCATCTTCTTCAAG  P22 GCTAGCGGATCTGACGGTTCA  F-circSR1 CS2  P23 TGCCCGAAGGCTACGTCCAGGTAAGTTTGATTCTTCAGAATTTTCTAGTTT  P24 GGTCTTTATTGTTTTCCAAGTTTTTTTTTTTTTTTGAGATGGAGTC  Fragment of F-circSR1 or F-circSR2 M2 plasmid  P25 CTTGGAAAACAATAAAGACCAAATCC  P26 CTGGACGTAGCCTTCGGGC  F-circSR2 CS1  P27 GAACCGTCAGATCCGCTAGCTTTTTTTGAGACGGAGTTTCACTCT  P20 TTGAAGAAGATGGTGCGCTCCTGTGGATGAGAAAACAGCTTAAAGC  F-circSR2 CS2  P28 TGCCCGAAGGCTACGTCCAGGTAGCTCTGAAATAATTTTCTCCTGG  P29 GGTCTTTATTGTTTTCCAAGCTCATGCCTGTAATCCCAGGAC  F-circSR1 or F-circSR2 M1  P30 GCTGTTTTCTCATCCACTTGAGCGCACCATCTTCTTC  P31 GAAGAAGATGGTGCGCTCAAGTGGATGAGAAAACAGC |
| F-circSR siRNA targeting sequence  siRNA-1 for F-circSR1 TGAAGACCATGGCTCCCTG  siRNA-2 for F-circSR1 AGCTTTGAAGACCATGGCT  siRNA-1 for F-circSR2 ATCTGATGAGACCATGGCT  siRNA-2 for F-circSR2 GCACATCTGATGAGACCAT |
| **F-circSR1 sequence**  ACCATGGCTCCCTGGCCTGAATTGGGAGATGCCCAGCCCAACCCCGATAAGTACCTCGAAGGGGCCGCAGGTCAGCAGCCCACTGCCCCTGATAAAAGCAAAGAGACCAACAAAAcagATAACACTGAGGCACCTGTAACCAAGATTGAACTTCTGCCGTCCTACTCCACGGCTACACTGATAGATGAGCCCACTGAGGTGGATGACCCCTGGAACCTACCCACTCTTCAGGACTCGGGGATCAAGTGGTCAGAGAGAGACACCAAAGGGAAGATTCTCTGTTTCTTCCAAGGGATTGGGAGATTGATTTTACTTCTCGGATTTCTCTACTTTTTCGTGTGCTCCCTGGATATTCTTAGTAGCGCCTTCCAGCTGGTTGGAGCTGGAGTCCCAAATAAACCAGGCATTCCCAAATTACTAGAAGGGAGTAAAAATTCAATACAGTGGGAGAAAGCTGAAGATAATGGATGTAGAATTACATACTATATCCTTGAGATAAGAAAGAGCACTTCAAATAATTTACAGAACCAGAATTTAAGGTGGAAGATGACATTTAATGGATCCTGCAGTAGTGTTTGCACATGGAAGTCCAAAAACCTGAAAGGAATATTTCAGTTCAGAGTAGTAGCTGCAAATAATCTAGGGTTTGGTGAATATAGTGGAATCAGTGAGAATATTATATTAGTTGGAGATGATTTTTGGATACCAGAAACAAGTTTCATACTTACTATTATAGTTGGAATATTTCTGGTTGTTACAATCCCACTGACCTTTGTCTGGCATAGAAGATTAAAGAATCAAAAAAGTGCCAAGGAAGGGGTGACAGTGCTTATAAACGAAGACAAAGAGTTGGCTGAGCTGCGAGGTCTGGCAGCCGGAGTAGGCCTGGCTAATGCCTGCTATGCAATACATACTCTTCCAACCCAAGAGGAGATTGAAAATCTTCCTGCCTTCCCTCGGGAAAAACTGACTCTGCGTCTCTTGCTGGGAAGTGGAGCCTTTGGAGAAGTGTATGAAGGAACAGCAGTGGACATCTTAGGAGTTGGAAGTGGAGAAATCAAAGTAGCAGTGAAGACTTTGAAGAAGGGTTCCACAGACCAGGAGAAGATTGAATTCCTGAAGGAGGCACATCTGATGAGCAAATTTAATCATCCCAACATTCTGAAGCAGCTTGGAGTTTGTCTGCTGAATGAACCCCAATACATTATCCTGGAACTGATGGAGGGAGGAGACCTTCTTACTTATTTGCGTAAAGCCCGGATGGCAACGTTTTATGGTCCTTTACTCACCTTGGTTGACCTTGTAGACCTGTGTGTAGATATTTCAAAAGGCTGTGTCTACTTGGAACGGATGCATTTCATTCACAGGGATCTGGCAGCTAGAAATTGCCTTGTTTCCGTGAAAGACTATACCAGTCCACGGATAGTGAAGATTGGAGACTTTGGACTCGCCAGAGACATCTATAAAAATGATTACTATAGAAAGAGAGGGGAAGGCCTGCTCCCAGTTCGGTGGATGGCTCCAGAAAGTTTGATGGATGGAATCTTCACTACTCAATCTGATGTATGGTCTTTTGGAATTCTGATTTGGGAGATTTTAACTCTTGGTCATCAGCCTTATCCAGCTCATTCCAACCTTGATGTGTTAAACTATGTGCAAACAGGAGGGAGACTGGAGCCACCAAGAAATTGTCCTGATGATCTGTGGAATTTAATGACCCAGTGCTGGGCTCAAGAACCCGACCAAAGACCTACTTTTCATAGAATTCAGGACCAACTTCAGTTATTCAGAAATTTTTTCTTAAATAGCATTTATAAGTCCAGAGATGAAGCAAACAACAGTGGAGTCATAAATGAAAGCTTTGAAG |
| **F-circSR2 sequence**  ACCATGGCTCCCTGGCCTGAATTGGGAGATGCCCAGCCCAACCCCGATAAGTACCTCGAAGGGGCCGCAGGTCAGCAGCCCACTGCCCCTGATAAAAGCAAAGAGACCAACAAAAcagATAACACTGAGGCACCTGTAACCAAGATTGAACTTCTGCCGTCCTACTCCACGGCTACACTGATAGATGAGCCCACTGAGGTGGATGACCCCTGGAACCTACCCACTCTTCAGGACTCGGGGATCAAGTGGTCAGAGAGAGACACCAAAGGGAAGATTCTCTGTTTCTTCCAAGGGATTGGGAGATTGATTTTACTTCTCGGATTTCTCTACTTTTTCGTGTGCTCCCTGGATATTCTTAGTAGCGCCTTCCAGCTGGTTGGAGATGATTTTTGGATACCAGAAACAAGTTTCATACTTACTATTATAGTTGGAATATTTCTGGTTGTTACAATCCCACTGACCTTTGTCTGGCATAGAAGATTAAAGAATCAAAAAAGTGCCAAGGAAGGGGTGACAGTGCTTATAAACGAAGACAAAGAGTTGGCTGAGCTGCGAGGTCTGGCAGCCGGAGTAGGCCTGGCTAATGCCTGCTATGCAATACATACTCTTCCAACCCAAGAGGAGATTGAAAATCTTCCTGCCTTCCCTCGGGAAAAACTGACTCTGCGTCTCTTGCTGGGAAGTGGAGCCTTTGGAGAAGTGTATGAAGGAACAGCAGTGGACATCTTAGGAGTTGGAAGTGGAGAAATCAAAGTAGCAGTGAAGACTTTGAAGAAGGGTTCCACAGACCAGGAGAAGATTGAATTCCTGAAGGAGGCACATCTGATGAG |
|  |
