# Supplementary material for: Circular RNA F-circSR derived from SLC34A2-ROS1 fusion gene promotes cell migration in non-small cell lung cancer
Source: Mol Cancer. 2019 May 22;18:98. doi: 10.1186/s12943-019-1028-9 (PMC6530145; doi:10.1186/s12943-019-1028-9)
Supplement: Supplementary file 2 — Figure S1. Absolute quantification of F-circSRs in HCC78 cells using qPCR. Figure S2. Characterization of cellular function of F-circSR in lung cancer cells. Figure S3. Validation of cellular function of F-circSR using pLaccase2 circRNA expression system. Figure S4. Predicted miRNA biding sites in F-circSRs. (DOCX 2870 kb) [file 12943_2019_1028_MOESM2_ESM.docx]

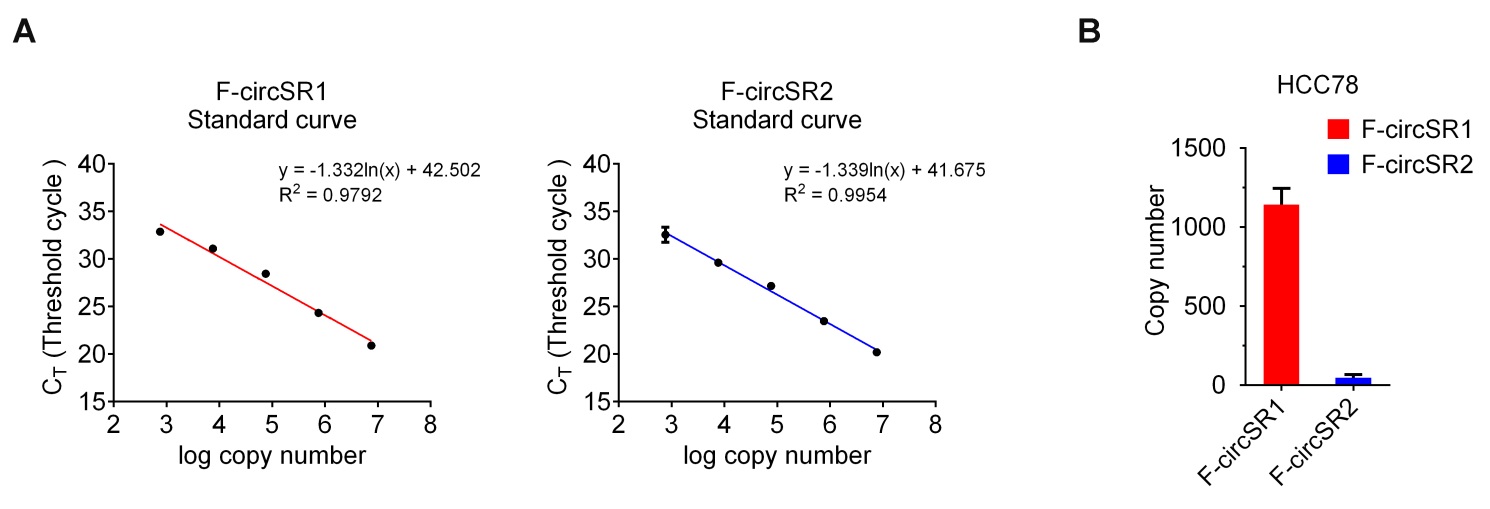


**Figure S1.** Absolute quantification of F-circSR in HCC78 cells. **(a)** Standard curves for F-circSR1 and F-circSR2. **(b)** Copy number of F-circSR1 and F-circSR2 in 1μg of random-primed HCC78 cDNA.


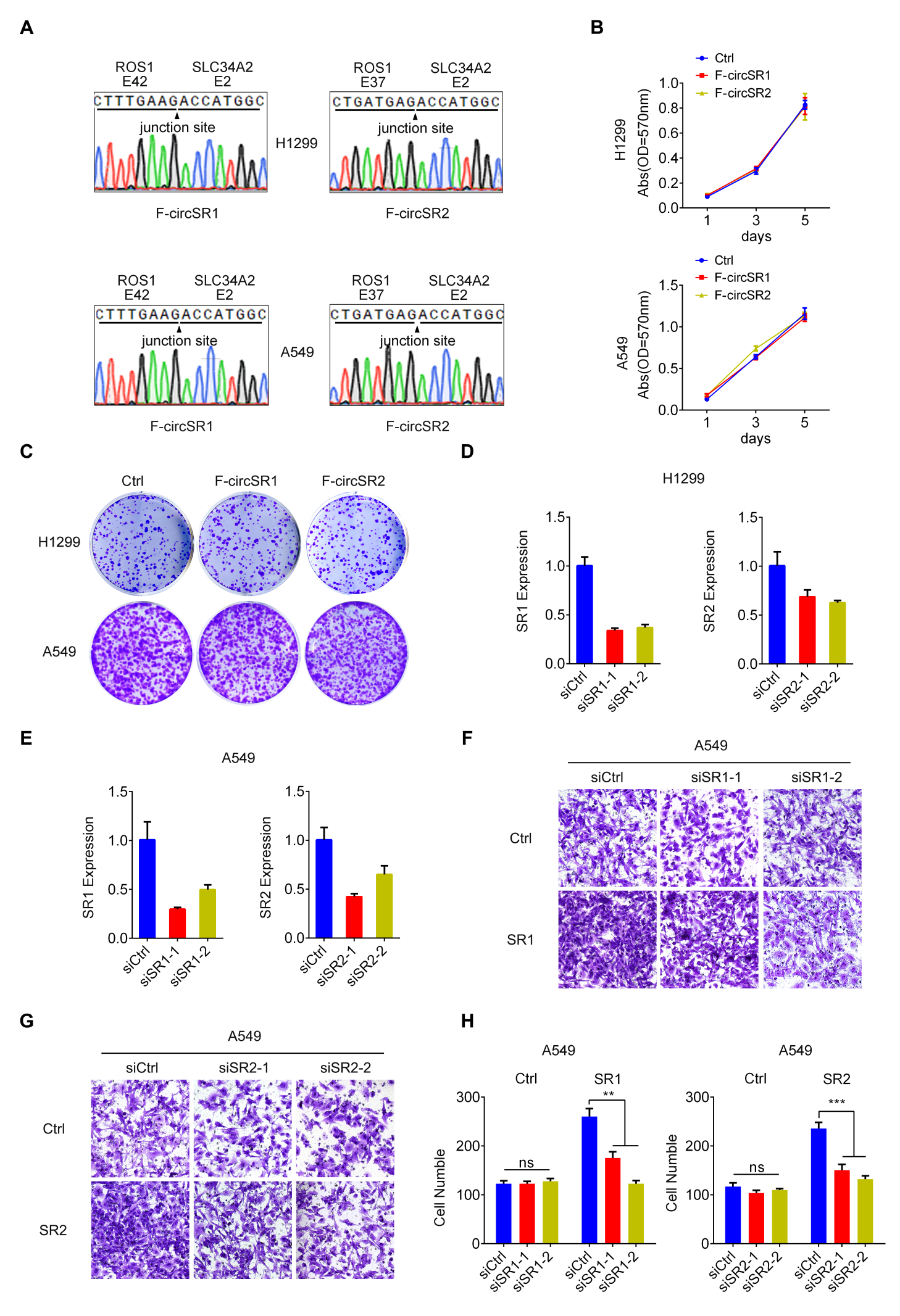


**Figure S2.** Characterization of cellular function of F-circSR in lung cancer cells. **(a)** Identification of accurate circulation of F-circSR by Sanger sequencing in stable H1299 and A549 cells overexpressing F-circSR. E: exon. **(b-c)** Effect of F-circSR on cell proliferation in H1299 and A549 cell, measured by MTT assays (b) and Colony formation assays (c). **(d-e)** RT-qPCR analysis showed effective knockdown of F-circSR by individual siRNA in F-circSR-overexpressing H1299 and A549 cells. SR1:F-circSR1; SR2:F-circSR2. **(f-h)** Knockdown of F-circSR1 (f) or F-circSR2 (g) attenuates the cell migratory ability in A549 cells stably overexpressing corresponding F-circSR. The migrated cell were counted and subjected to statistically analyzed (h) using Student t-test. ** *P* < 0.01, *** *P* < 0.001.


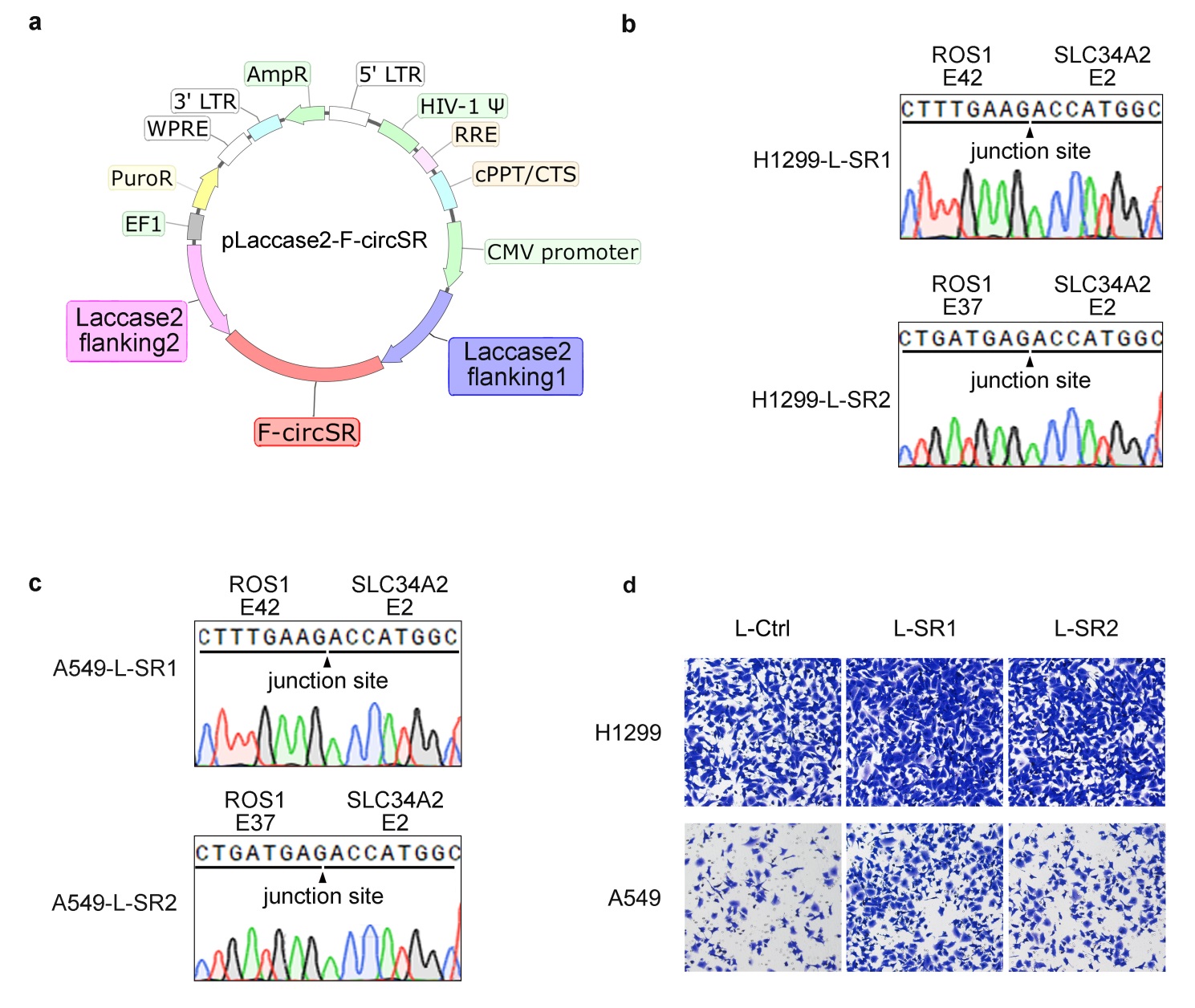


**Figure S3.** Validation of cellular function of F-circSR using pLaccase2 circRNA expression system. (**a**) Schematic representation of F-circSR-expressing plasmid in pLaccase2 vector. (**b-c**) Correct circulation of F-circSR in H1299 (b) and A549 cells (c) using pLaccase2 circRNA expression system. L:*laccase2*; SR1:F-circSR1; SR2:F-circSR2; E:exon. (**d**) Enhanced migratory ability by F-circSR overexpression in H1299 and A549 cell. Ctrl:control.


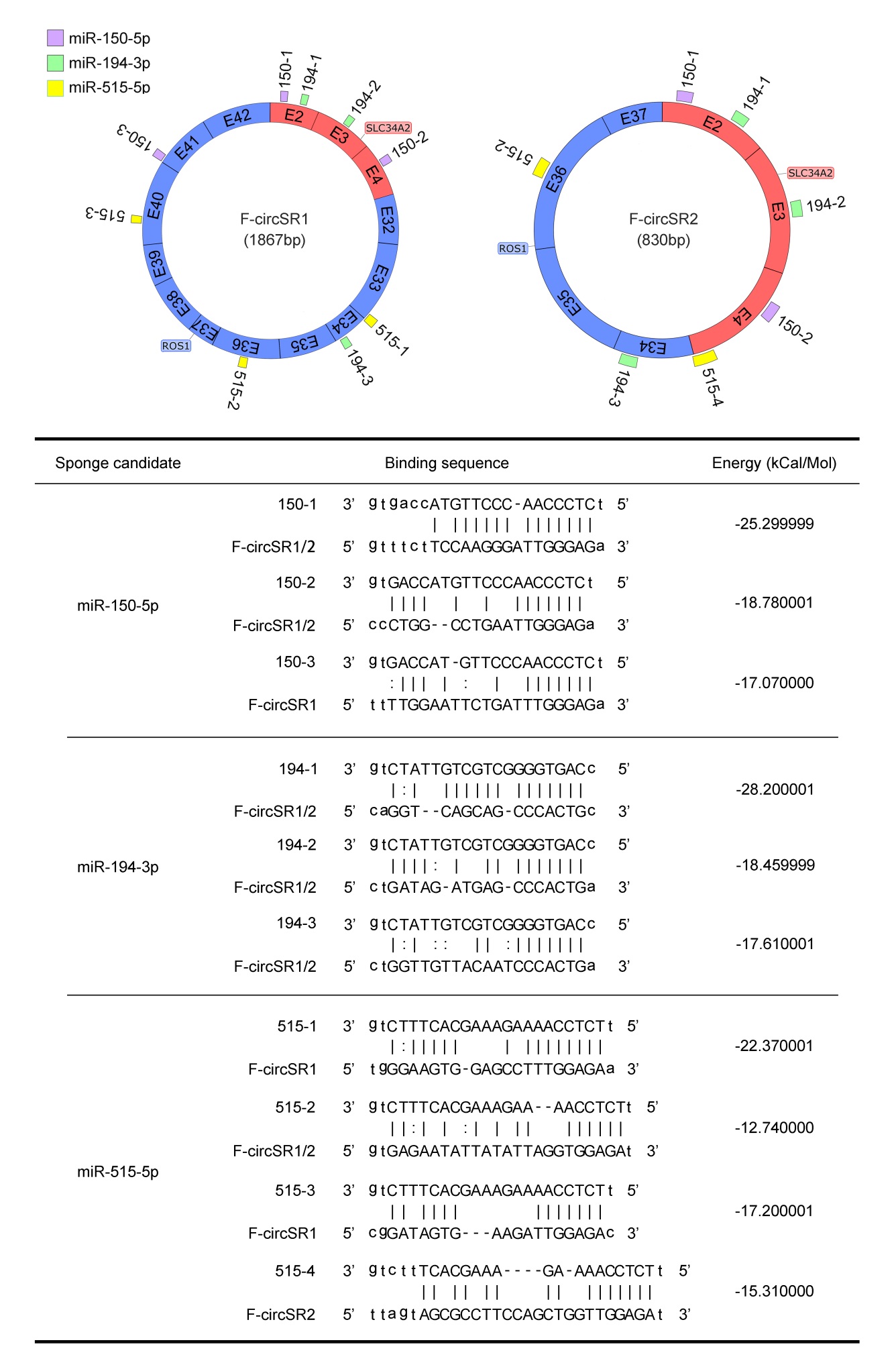


**Figure S4.** Prediction of potential miRNA binding sites in F-circSRs by miRanda program.
