# Supplementary material for: Circular RNA F-circSR derived from SLC34A2-ROS1 fusion gene promotes cell migration in non-small cell lung cancer
Source: Mol Cancer. 2019 May 22;18:98. doi: 10.1186/s12943-019-1028-9 (PMC6530145; doi:10.1186/s12943-019-1028-9)

Bioinformatics analysis of the cis-elements in the flanking introns of F-circSR

1. BLAST analysis of upstream sequence (CS1) with downstream sequence (CS2) of the F-circSR1 flanking introns


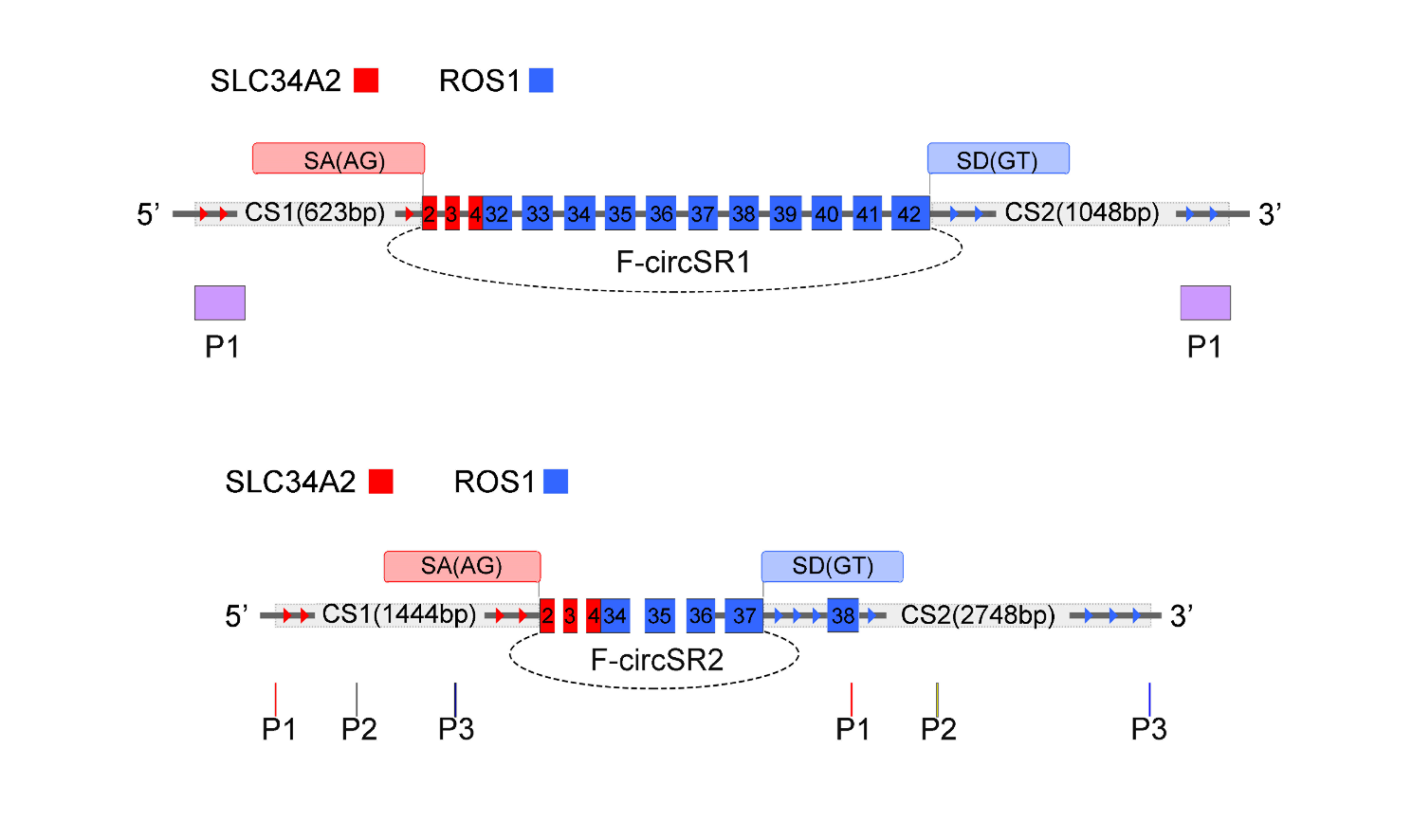


The red and blue boxes indicate the exons from SLC34A2 and ROS1 genes, respectively. The grey lines mean introns of SLC34A2-ROS1 fusion gene. The grey boxes indicate the upstream (CS1) and downstream sequences (CS2) of F-circSR1 used for bioinformatics analysis and GFP reporter assay; the boxes called P1 indicate the paired complementary bases identified by BLAST analysis. CS1: up-stream flanking sequence cloned into GFP reporter; CS2: down-stream flanking sequence cloned into GFP reporter; SA: splicing acceptor; SD: splicing donor; P: paired complementary bases.

Sbjct: the upstream sequence (CS1) of F-circSR1 flanking intron

Query: the downstream sequence (CS2) of F-circSR1 flanking intron

Paired complementary bases


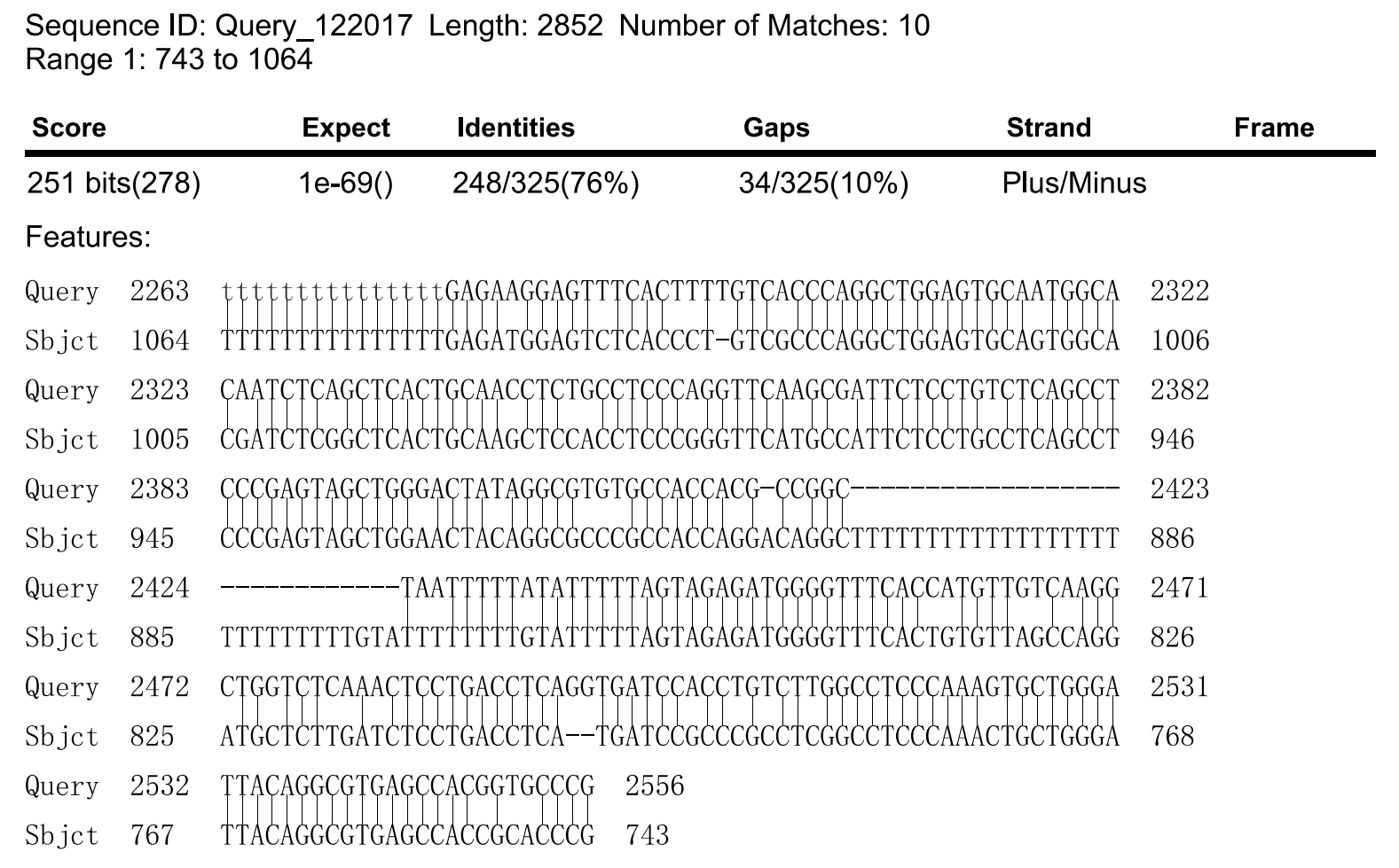


1. BLAST analysis of upstream sequence (CS1) with downstream sequence (CS2) of the F-circSR2 flanking introns


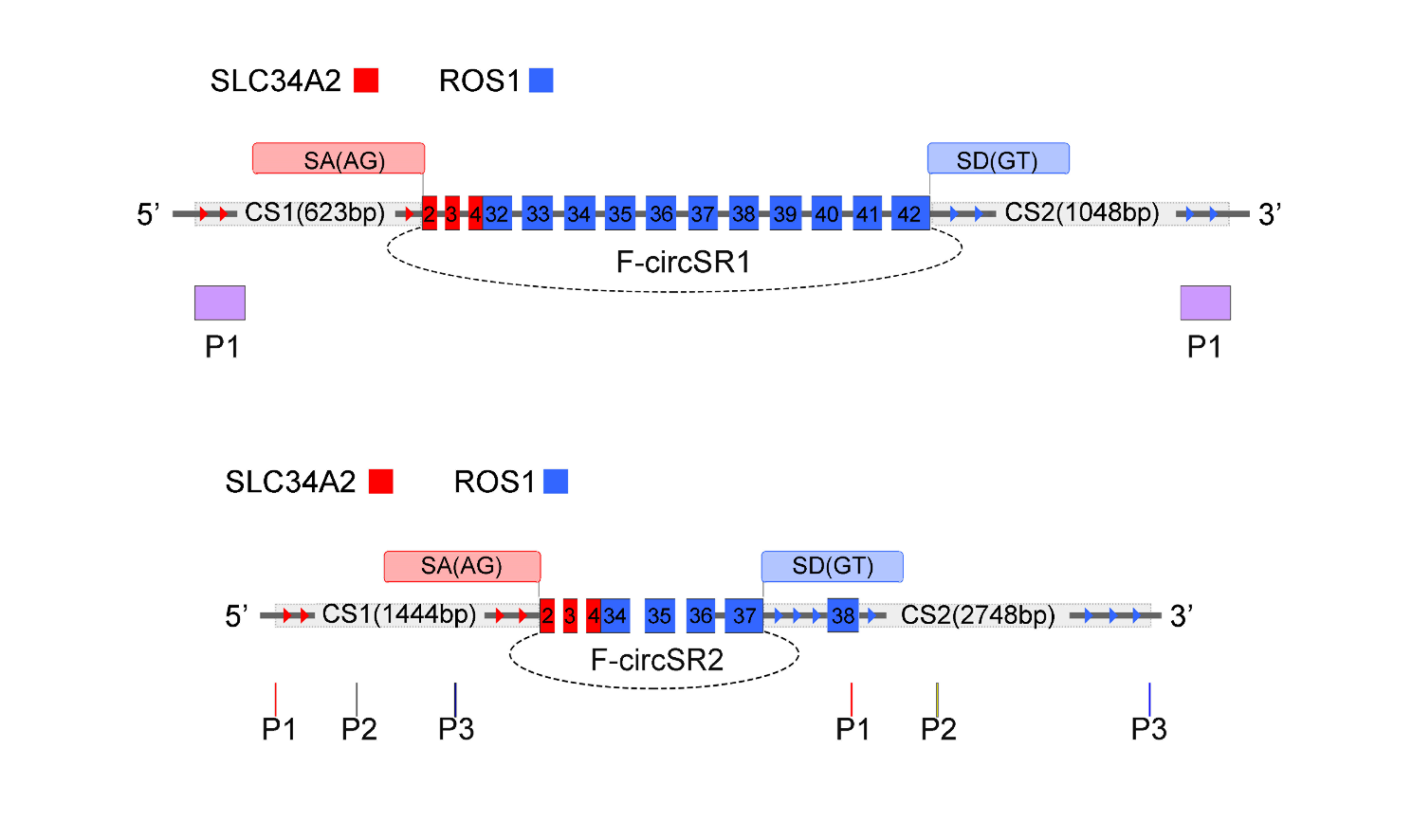


The red and blue boxes indicate the exons from SLC34A2 and ROS1 genes, respectively. The grey boxes indicate the upstream (CS1) and downstream sequences (CS2) of F-circSR2 used for bioinformatics analysis and GFP reporter assay; the boxes called P1, P2 and P3 indicate the paired complementary bases identified by BLAST analysis.

Sbjct: the upstream sequence (CS1) of F-circSR2 flanking intron

Query: the downstream sequence (CS2) of F-circSR2 flanking intron

Paired complementary bases 1:


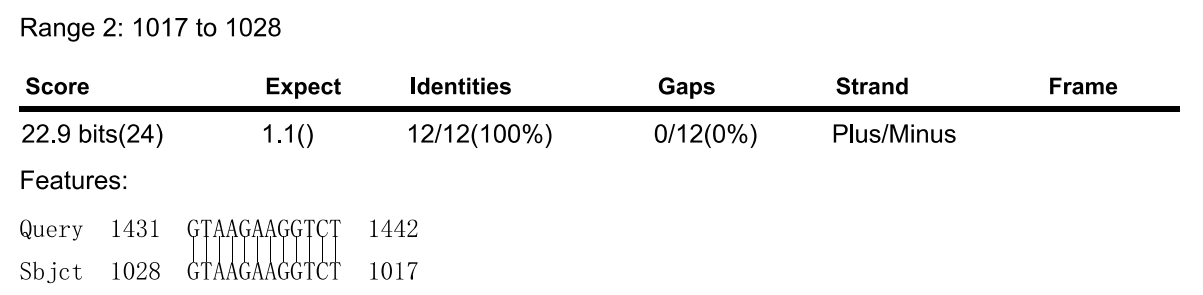


Paired complementary bases 2:


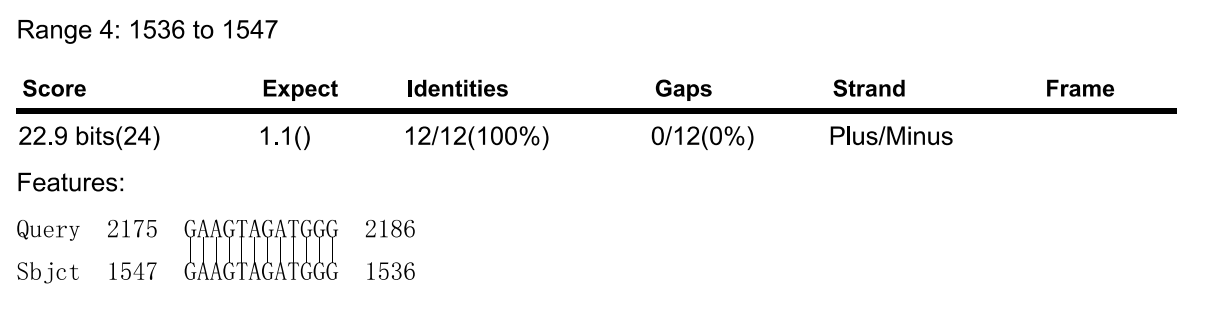


Paired complementary bases 3:


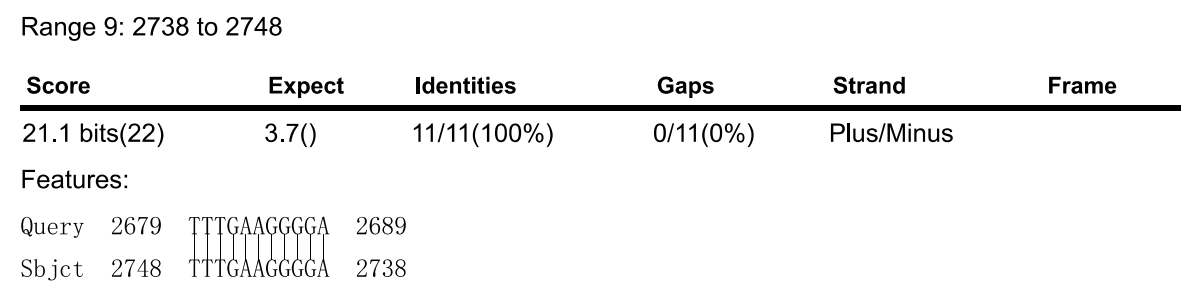

Supplement: Supplementary file 3 — Bioinformatics analysis of the cis-elements in the flanking introns of F-circSR. (DOCX 1084 kb) [file 12943_2019_1028_MOESM3_ESM.docx]
