# Supplementary material for: Circular RNA F-circSR derived from SLC34A2-ROS1 fusion gene promotes cell migration in non-small cell lung cancer
Source: Mol Cancer. 2019 May 22;18:98. doi: 10.1186/s12943-019-1028-9 (PMC6530145; doi:10.1186/s12943-019-1028-9)
Supplement: Supplementary file 4 — Supplementary Materials and Methods. (DOCX 2750 kb) [file 12943_2019_1028_MOESM4_ESM.docx]

# Supplementary Materials and Methods

## Plasmid construction

The circRNA expression plasmid pCRE5 was constructed by inserting the upstream flanking sequence of ciRS-7 and its reverse complementary sequence into the multiple cloning sites of pCDH-CMV-MCS-EF1-PURO (System Biosciences) by One Step Cloning Kit (Vazyme). For F-circSR overexpression, cDNAs reverse-transcribed from RNAs of HCC78 cells were used as PCR template and subjected to amplify the full length of F-circSR plus 50bp of upstream and downstream flanking intron sequences by two rounds of PCR, the resultants were inserted into the reverse repeat sequences of pCRE5 using One Step Cloning Kit (Vazyme).

Another circRNA expression plasmid pLaccase2 were constructed by inserting the core elements from the vector pcDNA3.1(+)-Laccase2-MCS-exon (generously provide by Prof. Jeremy E. Wilusz at University of Pennsylvania) for RNA circulation into the multiple cloning sites of pCDH-CMV-MCS-EF1-PURO (System Biosciences) by One Step Cloning Kit (Vazyme). Then the artificial exon of the inserted core elements was replaced by the sequence of F-circSR1 or F-circSR2 using One Step Cloning Kit (Vazyme).

The reporter plasmid pCIRC-FP-IRES-G for GFP is generously provided by Prof. Zefeng Wang (CAS-MPG Partner Institute for Computational Biology, Chinese Academy of Sciences). Inserts of IGF2BP1 intron 12 and its partial reverse complementary sequence within pCIRC-FP-IRES-G were replaced by CS1 and CS2 of F-circSR using One Step Cloning Kit (Vazyme), respectively. Briefly, the fragments were amplified from pCIRC-FP-IRES-G using primers P21/P22 to remove IGF2BP1 intron 12, and then recombined with CS1 fragments (amplified using primers P19/P20 for F-circSR1; P27/P20 for F-circSR2) to get the plasmid called M2 which lacking the complementary sequence. Then fragments amplified from the plasmid M2 using primers P25/P26 were recombined with CS2 fragments (amplified using primers P23/P24 for F-circSR1; P28/P29 for F-circSR2) to get the F-circSR normal plasmid. To construct M1 plasmid, sited-directed mutagenesis was performed using the primer P30/P31 to destroy the splicing acceptor sequence. All the primers used for cloning are listed in Additional file 1.

## RNA isolation, qPCR and circRNA identification

Total RNAs were extracted by TRIzol reagent (Life Technologies) and subjected to reverse transcription by random primers using the RETROscript kit (Life Technologies). Then the expression levels of target RNAs were measured with SYBR Green Master Mix using StepOnePlus^TM^ Real-Time PCR System (Applied Biosystems). *β*-actin was used as endogenous controls, and the fold change was calculated via the 2^-∆∆Ct^ method.

For circRNA identification, total RNAs (10 μg) were incubated with 30 units of Ribonuclease R (Epicentre Technologies) at 37°C for 1 hour to digest linear RNAs, followed by precipitation by ammonium acetate and glycogen (Invitrogen). The resultant RNAs were subjected to PCR amplification with divergent primers using Phanta Max Super-Fidelity DNA Polymerase (Vazyme). The primers used in this study are listed in Addition file 1.

## Absolute quantification of F-circSR levels in HCC78 cell

To better measure F-circSR level, divergent primers (P1/P2 for F-circSR1; P3/P4 for F-circSR2) were used for amplifying the fragment from cDNA of HCC78 cells crossing the junction site, and the resultant products were inserted into pMD19-T to obtain the standard plasmids (pMD19-T-F-circSR1 and pMD19-T-F-circSR2). The copy number of plasmid can be calculated as follows:

Weight in daltons (g/mol)= (bp size of DNA)×{(A+T)×AT%+(C+G)×GC%}

Hence: copy number$=\frac{DNA amount(g)\times Avogadro’s number (copy/mol)}{Weight in daltons (g/mol)}$

(where: bp = base pairs, A=312 Da, T=303 Da, C=288 Da, G=328 Da, AT%=AT content, GC%=GC content, Avogadro’s number=6.02×10^23^)

After serially 10-fold dilution, the diluted plasmids and HCC78 cDNA were added to subsequent real-time qPCR runs in the same reaction plate using the corresponding primer sets. For each diluted plasmid, determined C_T_ values were plotted versus the logarithm of their copy numbers. The standard curve was generated by linear regression through these points. The precise copy numbers of F-circSR were calculated from the corresponding standard curve, using the C_T_ value.

## Cell culture and construction of stable cells

NSCLC cells (H1299 and A549) were cultured with RPMI-1640 medium (Invitrogen) supplemented with 10% fetal bovine serum (Gibco) plus 100 U/mL penicillin/streptomycin (Sigma). All cells were maintained at 37°C in a humidified incubator with 5% CO_2_.

For lentivirus preparation, HEK293T cells were transfected using Lipofectamine 2000 (Invitrogen) with the lentiviral vector pCRE5 or pLaccases2, packaging plasmid pCMV-dR8.2 dvpr (System Bioscience), and envelope plasmid pCMV-VSVG (System Bioscience). At 72 hours after infecting the cells, the stable cells were selected by 2 μg/mL of puromycin.

## Transwell Cell migration assays

Cell migration assays were performed using Transwell chamber (Millipore), RPMI-1640 medium supplemented with 10% FBS was added into the bottom chambers, then cells were suspended with RPMI-1640 medium containing 5% BSA and seeded into the top chamber. After incubating at 37°C for 20 hours, cells that did not migrate through the pores were removed with a cotton swab. The Transwell chambers were put into 4% paraformaldehyde for 30 min, followed by staining with 1% crystal violet for 30 min. The cells on the bottom of the chamber were counted with an inverted phase-contrast microscope (at least three randomly selected fields).

## Wound healing assay

Cells were cultured in 6-well plate for 12h and then wounded by a sterilized pipet tip to make a straight scratch. After being washed with PBS gently, cells were cultured in RPMI-1640 medium with 0.5% FBS and 1% penicillin/streptomycin. Pictures were taken by an Olympus digital camera at 0 hour and 24 hours after wounding.

## Colony formation and MTT assays

For colony formation assay, 3,000 cells were seeded into 6-well plate and cultured for 7 days. The cells were fixed with 4% paraformaldehyde for 30 min, then stained with 1% crystal violet (Sigma) for 30 min. The cells were washed to remove crystal violet solution with water and colony formation were recorded by a high-resolution scanner.

For MTT assays, 1,000 cells per well were seeded into 96-well plate for MTT assay. The cells were incubated with 0.5 mg/mL filtered sterile MTT (Beyotime) at 37℃ for 4 hours at the indicated time point, then the media were removed and replaced with 150 μL DMSO and then measure the absorbance at 570 nm.

## Prediction of miRNA binding sites in F-circSR

Computer program miRanda (v3.3a) was used to search the potential binding sites of human miRNAs within F-circSR according to the following cut-off parameters: miRanda score >100, minimal free energy of binding less than -12 kCal/Mol, alignment with the seed region of miRNA, the gap-opening penalty to -8 and the gap-extend penalty to -2 for alignments.
